# Supplementary material for: Hidden Treasures of Colombia’s Pacific Mangrove: New Fungal Species and Records of Macrofungi (Basidiomycota)
Source: J Fungi (Basel). 2025 Jun 17;11(6):459. doi: 10.3390/jof11060459 (PMC12194073; doi:10.3390/jof11060459)
Supplement: Supplementary file 1 [file jof-11-00459-s001.zip › jof-3617164-supplementary.pdf]

Table S1. List of sequences generated in this study and accession numbers retrieved from the NCBI. For each collection, the species name, voucher, locality, reference, and GenBank accession number are provided. Type specimens are denoted with an asterisk (*). Missing information is indicated with an n-dash (–). New species sequences are indicated in Bold.

Specimen	Voucher	Locality	GenBank Accession No.		
			ITS	nLSU	References
Fomitopsis **P. Karst. phylogeny**					
F. bambusae Y.C. Dai, Meng Zhou & Yuan Yuan (T)	Dai 22116	CN	MW937876	MW937883	[1]
	Dai 22114	CN	MW937875	MW937882	[1]
F. caespitosa (Murrill) Spirin & Miettinen	Cui 10140 (BJFC)	CN	JQ067651	JX435771	[2]
	Miettinen 13076	ID	KC595912	KC595912	[3]
F. eucalypticola B.K. Cui & Shun Liu	Cui 16594	AU	MK852560	MK860110	[4]
	Cui 16598	AU	MK852562	MK860113	[4]
F. luzonensis (Murrill) Spirin & Miettinen	Miettinen 14311	ID	KC595920	KC595920	[3]
	Miettinen 13163	ID	ON970638	ON970638	[5]
F. marianiae (Bres.) Spirin, Vlasák & Cartabia	TYP 6137	JP	KJ995920	–	Unpublished
	Rivoire 6563	FR	ON754051	–	[5]
	Spirin 5176	RU	ON754053	–	[5]
F. meliae (Underw.) Gilb.	Ryvarden 16893	–	KR605776	KR605715	[6]
	FM1C33	US	MW567243	–	Unpublished
	Vlasak 1704/78J (H)	CR	ON994706	–	[5]
F. nivosella (Murrill) Spirin & Vlasák	**ACB1573**	**CO**	**PV330456**	–	**This study**
	**ACB1580**	**CO**	**PV330457**	**PV299508**	**This study**
	**ACB1587**	**CO**	**PV330458**	**PV299509**	**This study**
	**ACB1654**	**CO**	**PV330459**	**PV299510**	**This study**
	**ACB1660**	**CO**	**PV330460**	**PV299511**	**This study**
	**ACB1675**	**CO**	**PV330461**	**PV299512**	**This study**
	De Jesus OF10833	BR	ON994715	–	[5]

	De Meijer 3465	BR	ON994716	–	[5]
	Kout 180719	US	ON994717	–	[5]
F. ostreiformis (Berk.) T. Hatt.	Miettinen 10071 (H)	CN	KC595914	KC595914	[3]
	Miettinen 88541 (H)	ID	ON970636	ON970636	[5]
F. palustris (Berk. & M.A. Curtis) Gilb. & Ryvarden	Dollinger 782	US	ON994721	–	[5]
	Lowe 4092	US	ON994722	–	[5]
	Ryvarden 44439	BZ	ON994723	–	[5]
**ROOT:** *F. pinicola* (Sw.) P. Karst.	AFTOL ID770	RU	AY854083	AY684164	Unpublished
***Lentinus* Fr. phylogeny**					
L. arcularius (Batsch) Zmitr.	Dai 8159	–	KC572005	KC572044	[7]
	Cui 10998	CN	KX548973	KX548995	[7]
L. badius (Berk.) Berk.	JS0094	MY	KP283478	KP283512	[8]
	DED 07668	TH	KP283480	KP283518	[8]
L. brumalis (Pers.) Zmitr.	Cui 10750	CN	KU189765	KU189796	[7]
	EP4	US	KP283490	KP283519	[8]
L. crinitus (L.) Fr.	Roland EC795	EC	GU207297	–	[9]
	Roland 35436	VE	GU207292	KP283523	[9]
	DSH9243C	CR	KP283495	KP283523	[8]
L. longisporus (Audet, Boulet & Sirard) Zmitr. & Kovalenko	WD 2579	JP	AB478879	LC052218	[10]
	DAOM 229479	CA	AB478880	LC052217	[10]
L. polychrous Lév.	KM141387	TH	KP283487	KP283514	[8]
	JS 00054	MY	KP283486	KP283523	[8]
L. sajor-caju (Fr.) Fr.	SNP 24989	BR	KP283493	KP283510	[8]
	JS0056	MY	KP283494	KP283511	[8]
Lentinus scleropus (Pers.) Fr.	**ACB1507**	**CO**	**PV330462**	**PV299513**	**This study**
	TFB 11164	MX	GU207310	–	[9]
	TH548	FG	PP102338	–	Unpublished

L. squarrosulus Mont.	Cui 6513	CN	KP283482	KP283516	[8]
	BORH 0009	MY	KP283484	KP283515	[8]
L. substrictus Lloyd	TENN 10167	DK	AB070883	AJ487943	[11]
	Wei 1582	CN	KU189767	KU189798	[7]
	TUFC 101630	CN	LC486771	LC486773	[12]
L. tigrinus (Bull.) Fr.	DSH 92D787	US	KP283488	–	[8]
	MUCL 22821	BE	–	AB368072	[10]
**ROOT:** *L. tricholoma* (Mont.) Zmitr. (ROOT)	Cui 8468	CN	JX559277	JX559302	[13]
***Microporus* P. Beauv. phylogeny**					
M. affinis (Blume & T. Nees) Kuntze	MEL:2382659	AU	KP013022	KP013022	Unpublished
	MEL:2382709	AU	KP012889	KP012889	Unpublished
	**ACB1512**	**CO**	**PV330463**	**PV299514**	**This study**
M. flabelliformis (Fr.) Pat.	JBRI–M23-002	–	PQ613372	–	Unpublished
	DCY2498	CN	MZ666431	–	Unpublished
	Dai 11574	CN	JX569740	JX569747	Unpublished
M. vernicipes (Berk.) Kuntze	CLZhao 5612	CN	ON319076	–	Unpublished
	KUC20130711-23	SK	KJ668503	KJ668355	Unpublished
	KUC11046	SK	KJ714006	–	[14]
	Dai7252	CN	KX880619	–	Unpublished
	Dai9283	CN	KX880618	KX880658	Unpublished
M. xanthopus (Fr.) Kuntze	Dai12076	CN	KX880620	KX880659	Unpublished
	PEN79	MY	LC471202	AB368075	[15]
**ROOT:** *Lentinus flexipes* (Fr.) Zmitr. & Kovalenko	X2185	CN	PP660620	PP658229	Unpublished
***Neohypochnicium* N. Maek. & R. Sugaw phylogeny**					
N. albostramineum (Bres.) N. Maek.	FCUG1772	SE	AF429423	–	[16]
	FCUG269	SE	AF429422	–	[16]
	FCUG1865	SP	AF429421	–	[16]

N. aoteraroae (B.C. Paulus, H. Nilson & Hallenb.) N. Maek.	FCUG2972	NZ	DQ309071	–	[17]
	FCUG3120	NZ	GQ906536	–	[18]
N. asiaticum N. Maek. & Kogi (T)	TUMH: 61220	JP	LC663669	LC663689	[19]
	TUMH: 61227	JP	LC663672	–	[19]
	TUMH: 64610	JP	LC663673	–	[19]
N. cremicolor (Bres.) N. Maek.	FCUG160	DN	AF429425	–	[17]
	CBS:208.54	FR	MH857294	MH868826	[20]
	LWZ2021092121a	CN	ON897885	ON885344	Unpublished
N. cystidiatum (Boidin & Gilles) N. Maek.	FCUG3086	CF	DQ658163	–	[17]
	FCUG3087	GA	DQ658164	–	[17]
N. geogenium (bres.) N. Maek.	FCUG2052	FR	AF429426	–	[16]
	UC2023004	US	KP814198	–	Unpublished
	MA–Fungi 48308	SE	FN552534	JN939576	[18]
	He6804	CN	OM039279	OM039179	Unpublished
	He6817	CN	OM039281	OM039181	Unpublished
	He6819	CN	OM039282	OM039182	Unpublished
	HHB11137sp	US	KY948800	–	[21]
	NH 10910 (GB)	SE	DQ677509	DQ677509	[22]
	F-777	SE	PQ653225	PQ653225	Unpublished
	CLZhao 18234	CN	PP515411	–	Unpublished
	CLZhao 18093	CN	PP515410	–	Unpublished
	HHB9373sp	US	KY948799	–	[21]
	olrim193	SE	AY805611	–	[23]
N. guineense (Telleria, M. Dueñas, Melo & M.P. Martin) N. Maek. (T)	MA:Fungi-79156	GQ	FN552536	–	[18]
N. huinayense (Telleria, M. Dueñas, Melo & M.P. Martin) N. Maek. (T)	MA-Fungi 19598	CH	HG000303	–	[19]
	MA-Fungi 13980	CN	HG326616	–	[19]
***N. manglarense* sp. nov.**	**ACB1490***	**CO**	**PV330464**	–	**This study**
N. michelii (Telleria, M. Dueñas, Melo & M.P. Martin) N. Maek. (T)	MA:Fungi 79155	SP	FN552535	JN939579	[18]

N. patagonicum (Gorjón & Hallenb.) N. Maek.	GB-0129149	CH	HG000304	–	[19]
N. perlongicystidiosum N. Maek., Kogi & Norikura (T)	TUMH:63328	JP	LC662676	–	[19]
	TUMH:40397	JP	LC663679	LC663690	[19]
N. pini (Y. Jang & J.J. Kim) N. Maek.	F0023765	CN	KC282472	–	[24]
	TUMH: 61221	JP	LC663680	LC663691	[19]
	TUMH: 64587	JP	LC663681	–	[19]
	NIBRFG0000107453	KR	JX217823	–	[24]
N. punctulatum (Cooke) N. Maek.	FCUG1203	NO	AF429412	–	[16]
	FCUG1921	DK	AF429410	–	[16]
	TUMH61188	US	LC663682	–	[19]
	FP101698sp	US	KY948827	KY948860	[21]
N. subrigescens (Boidin) N. Maek.	TUMH: 64612	JP	LC663685	LC663692	[19]
	KHL11968-GB	NO	JQ031128	JQ031128	[25]
	TUMH: 61539	JP	LC663686	–	[19]
	FCUG1966	DK	AF429427	–	[18]
	RLG-15138	US	KY948803	KY948861	[21]
N. wakefieldiae (Bres.) N. Maek.	MA:Fungi-7675	SP	FN552531	JN939577	[18]
	FCUG1709	FI	AF429419	–	[16]
	FCUG2194	FI	AF429415	–	[16]
**ROOT:** *Abortiporus biennis* (Bull.) Singer	CBS:676.70	US	MH859896	MH871686	[20]
***Oudemansiella* Speg. phylogeny**					
O. australis G. Stev. & G.M. Taylor	RV95/297	AU	AF321472	–	[26]
	RV95/416	AU	AF321473	–	[26]
O. canarii (Jungh.) Höhn.	CAG5	PH	OR083770	–	Unpublished
	OS1	PH	OR052680	–	Unpublished
	HKAS38350	CN	AY436426	AY804259	Unpublished
	JM98/221	CN	AF321476	–	[26]
O. crassifolia Corner	HKAS38350	CN	AY665205	AY804278	Unpublished

O. cubensis (Brk. & M.A. Curtis) R. H. Petersen	RVPR33	CR	AF321478	–	[26]
	RVPR100	PR	AF321479	AF261351	Unpublished
	TENN59771	DR	GQ892791	–	Unpublished
	TENN49023	PR	GQ892792	HM005115	Unpublished
	TENN56534	CR	GQ892790	–	Unpublished
	RV96/35	CR	AF321477	–	[26]
	TENN58954	AR	GQ892789	–	Unpublished
	ECO-TA-HO 7876	MX	MF156259	–	Unpublished
O. platensis (Speg.) Speg.	**ACB1478**	**CO**	**PV330465**	**PV299515**	**This study**
	**ACB1571**	**CO**	**PV330467**	**PV299517**	**This study**
	TENN62802	US	GQ892793	–	Unpublished
	FLAS-F-61207	US	MH211812	–	Unpublished
	TENN51190	CR	GQ892794	–	Unpublished
	14v	AR	MT272111	–	Unpublished
	15v	AR	MT2	–	Unpublished
	3326	AR	MT272115	–	Unpublished
	33v	AR	MT272113	–	Unpublished
**ROOT:** *Xerula pudens* (Pers.) Singer	FPopa1969	DE	MF063189	MF063124	[27]
***Paramarasmius* Antonín & Kolarík phylogeny**					
Chaetocalathus liliputianus (Mont.) Singer	BRNM 751596	–	KF380834	KF380838	[28]
Chaetocalathus conchatus (Har. Takah.) Vizzini	C6186	EC	AY571032	AY570996	[29]
Moniliophthora roreri (Cif.) H.C. Evans, Stalpers, Samson & Benny	CBS 202.77	EC	MH861051	MH872819	[20]
Moniliophthora perniciosa (Stahel) Aime & Phillips-Mora	COAD 2612	BR	MK785154	MK785250	Unpublished
Paramarasmius mesosporus (Singer) Antonín, K. Hosaka & kolarík (T)	TNS-F-48339	JP	OM522625	OM522623	[30]
	MCVE 15643	IT	OM522629	–	[30]
	BRNM 828732	KR	OM522626	OM522619	[30]
	RSG 4FA	CN	MF356590	–	[31]
Paramarasmius palmivorus (Sharples) Antonín & Kolarík	**ACB1519**	**CO**	**PV330467**	**PV299517**	**This study**

	MMPS30	MY	MN871736	MN934819	Unpublished
	GH-11	GH	MN794131	MN794066	Unpublished
	CMU-NK076	TH	MW647877	MW647892	Unpublished
	AKD112/215	IN	MG251431	MG251441	Unpublished
**ROOT:** *Marasmius guyanensis* Mont.	JO344	--	MN714048	OR656978	[32]
***Phlebiopsis* Jülich phylogeny**					
P. alba (G. Cunn.) Stalpers	He 5432	CN	MT386391	MT447428	Unpublished
	GC 1508-10	TW	MZ637042	MZ637246	[33]
P. albescens Y.N. Zhao & S.H. He	He 5805*	CN	MT452526	–	[34]
P. amethystea (Hjortstam & Ryvarden) R.S. Chikowski & C.R.S. de Lira	CL161	BR	MK993644	MK993638	[35]
	URM84741	BR	MK993645	MK993639	[35]
	TRF307	BR	MK993647	MK995634	[35]
	CL1891	BR	MK993648	–	[35]
	**MV534**	**BR**	**PV562813**	**PV562973**	**This study**
	**AL93**	**BR**	**PV562814**	**PV562974**	**This study**
	**MV474**	**BR**	**PV562815**	**PV562975**	**This study**
P. brunnea Y.N. Zhao & S. H. He	He 5822*	CN	MT452527	MT447451	[34]
P. brunneocystidiata (Sheng H. Wu) Miettinen	Chen 666*	CN	MT561707	GQ470640	Unpublished
P. castanea (Lloyd) Miettinen & Spirin	Spirin 5295 (H)*	RU	KX752610	KX752610	[36]
	GC 1612-6	TW	KY688208	–	[33]
***P. colombiana* sp. nov.;l.**	**ACB1508***	**CO**	**PV330468**	**PV299518**	**This study**
	**MV396**	**BR**	**PV562816**	**PV562976**	**This study**
	**MV650**	**CO**	**PV562817**	**–**	**This study**
	**ACB1655**	**CO**	**PV562818**	**–**	**This study**
	103 F9C-AM	BR	MG751231	–	Unpublished
	FLASF61132	US	MH211764	–	Unpublished
P. crassa (Lév.) Floudas & Hibbett	He 3349	CN	MT561712	MT447407	[34]
	He 6304		MT561714	MT598029	Unpublished

	KKN-86	AR	KP135394	KP135215	[37]
	HHB 8834	US	KP135393	–	[37]
	**MV 397**	**BR**	**PV562819**	**PV562977**	This study
	**MV 442**	**BR**	**PV562820**	–	This study
P. cylindrospora Y.N. Zhao & S.H. He	He 5984*	CN	MT386404	MT447445	[34]
	He 5932	CN	MT386403	MT447444	[34]
P. daweishanensis J.H. Dong & C.L. Zhao	CLZhao 17984*	CN	OR096193	OR461451	[38]
P. fisurata Y.L. Deng & C.L. Zhao	CLZhao 30147*	CN	OR917877	OR921223	[38]
	CLZhao 30247	CN	OR917878	OR921226	[38]
P. flavidoalba (Cooke) Hjortstam	**ACB1495**	**CO**	**PV330469**	**PV299519**	**This study**
	Miettinen 17896 (H)	US	KX752607	KX752607	[36]
	HHB-4617	US	KP135401	–	[37]
	FD-263	US	KP135402	KP135271	[37]
	MC1	US	PP715425	–	Unpublished
	SH 52	MX	OQ955295	OQ955294	Unpublished
	Miettinen 17897	US	KP135398	–	[37]
P. friesii (Lév.) Spirin & Miettinen	He5722	LK	MT452528	MT447413	[34]
	He 5817	CN	MT452529	MT447414	[34]
P. galochroa (Bres.) Hjorstam & Ryvarden	FP-102937-Sp	PR	KP135391	–	[37]
P. gigantea (fr.) Jülich	CBS: 935.70	DE	MH860011	MH871798	[20]
	FP-70857-Sp	US	KP135390	KP135272	[37]
	HHB 11416-Sp	US	KP135388	–	[37]
P. lacerata C.L. Zhao (T)	SWFC00003692*	CN	MT180946	MT180950	Unpublished
	SWFC00003705	CN	MT180947	MT180951	Unpublished
P. lamprocystidiata (Sheng H. Wu) Sheng H. Wu & Hallenb.	He 5910	CN	MT386383	MT386383	[34]
	He 3874	CN	MT386382	MT447418	[34]
P. laxa (Sheng H. Wu) Miettinen (T)	Wu 9311-17*	CN	MT561710	GQ470649	[39] [40]

P. membranacea Y.N. Zhao & S.H. He (T)	He 3849*	CN	MT386401	MT447441	[34]
	He 3842	CN	MT386400	MT447440	[34]
P. odontoidea C.C. Chen & Sheng H. Wu (T)	GC 1708-181*	CN	MZ637054	MZ637255	[33]
	GC 1708-182	CN	MZ637055	MZ637256	[33]
P. pilatii (Parmasto) Spirin & Miettinen	He 5114	CN	MT386385	MT447421	[34]
	Spirin 5048 (H)	RU	KX752590	KX752590	[36]
P. ravenelii (Cooke) Hjorstam	CBS: 411.50	FR	MH856691	MH868208	[20]
	FP-110129-Sp	US	KP135362	KP135274	[37]
P. sinensis Y.N. Zhao & S.H. He (T)	He 4673*	CN	MT386397	MT447435	[34]
	He 4295	CN	MT386395	MT447433	[34]
P. yunnanensis C.L. Zhao	CLZhao 3958	CN	MH744140	MH744142	[34]
	He 2623	CN	MT386387	MT447423	[34]
P. yushaniae C.C. Chen & Sheng H. Wu	Chen 2358*	TW	MZ637047	MZ637261	[33]
	Chen 1914	TW	MZ637045	–	[33]
**ROOT:** *Phaeophlebiopsis peniophoroides* (Gilb. & Adask.) Floudas & Hibbett	FP-150577	US	KP135417	KP135273	[37]
***Porogramme* (Pat.) Pat. phylogeny**					
P. albocincta (Cooke & Massee) Gibertoni	PR1478R	PR	KY948724	–	[21]
	PR1478T	PR	KY948725	KY948838	[21]
P. aurantiaca (A.M.S. Soares) Y.C. Dai, W.L. Mao & Yuan Yuan	Dai 17401	BR	ON261666	ON261637	[41]
	WX2014-115	BR	MH842137	MH844886	Unpublished
P. austroasiana Y.C. Dai, W.L. Mao & Yuan Yuan	Dai 19624	LK	ON261668	ON261639	[41]
	Dai 19634	LK	ON261669	ON261640	[41]
***P. bononiae* sp. nov.**	**ACB1486**	**CO**	**PV330470**	**PV299520**	**This study**
	**ACB1494***	**CO**	**PV330471**	**PV299521**	**This study**
P. brasiliensis (Ryvarden) Y.C. Dai, W.L. Mao & Yuan Yuan	WX2014-28	BR	MH844866	MH844865	Unpublished
	WX2014-100	BR	MH844679	MH844583	Unpublished
	**ACB1510**	**CO**	**PV330472**	**PV299522**	**This study**

P. bubalina (H.S. Yuan) Y.C. Dai, W.L. Mao & Yuan Yuan	Yuan 5801	CN	JQ319495	–	[42]
	Yuan 5813	CN	JQ319499	–	[42]
P. cylindrica Y.C. Dai, W.L. Mao & Yuan Yuan	Dai 18526A	CN	ON261671	ON261641	[41]
	Dai 18529A	CN	ON261672	ON261642	[41]
P. epimiltina (Bark. & Broom) Y.C. Dai, W.L. Mao & Yuan Yuan	Dai 19483	LK	OP997538	OP997547	[41]
	Dai 19625	LK	OP997539	OP997548	[41]
P. hinnulea (H.S. Yuan) Y.C. Dai, W.L. Mao & Yuan Yuan	Dai 13664	CN	OP997540	OP997549	[41]
	Yuan 5832	CN	JQ319500	–	[42]
P. micropora (A.M.S. Soares & W.K.S. Waxier) Y.C. Dai, W.L. Mao & Yuan Yuan	FP102875sp	PR	KY948726	–	[21]
	WX2014-116	BR	MH842144	–	Unpublished
	**MV955**	**BR**	**PV562821**	**PV562978**	**This study**
P. subargentea (Speg.) Y.C. Dai, W.L. Mao & Yuan Yuan	Dai 17445	BR	ON261675	ON261645	[41]
	Dai 17460	BR	ON261676	ON261646	[41]
	WX2014-26	BR	MH819426	MH842138	Unpublished
P. venezuelica (Ryvarden) Y.C. Dai, W.L. Mao & Yuan Yuan	O-F-76258	VE	MT216233	–	Unpublished
P. yunnanensis Y.C. Dai, W.L. Mao & Yuan Yuan	Dai 12,222	CN	KF913423	KF913427	Unpublished
	Dai 12,259	CN	KF913424	KF913428	Unpublished
**ROOT:** *Cyanoporus* aff. *fuligo* (Berk. & Broome) Y.C. Dai, W.L. Mao & Yuan Yuan	FP150657	BZ	KY948716	KY948840	[21]
***Punctularia* Pat. phylogeny**					
P. atropurpurascens (Berk. & Broome)	Dai 22678	CN	OL457970	OL457440	Unpublished
	WEI 17-662	–	MW570883	MW570888	[43]
	HM1	MX	OL840322	–	[44]
	FDS-CA-03289	US	PQ140071	–	Unpublished
	UDB039800	IT	UDB039800	–	Unpublished
	UNITE114604	IT	UDB035018	–	Unpublished
P. bambusicola C.L. Zhao	CLZhao 9098*	CN	MW559983	MW559985	[45]
	CLZhao 4133	CN	MW559982	MW559984	[45]

P. strigosozonata (Schwein.) P.H. Talbot	**ACB 1592**	**CO**	**PV330473**	**PV299523**	[45]
	CBS:345.34	–	MH855559	MH867064	[20]
	CIRM-BRFM 694	FR	PV109543	–	Unpublished
	He 4980	CN	MW507073	MW507013	Unpublished
	FH: BHI-F586	US	MH558554	–	Unpublished
	CFMR:RF7JR	US	KU668972	–	Unpublished
	HHB-11897-sp	US	DQ398958	AF518642	Unpublished
	UDB0 16433	EE	UDB016433	–	Unpublished
P. subhepatica (Berk.) Hjortstam	He 4436	CN	MW507074	MW507014	Unpublished
	He 5695	CN	MW507075	MW507015	Unpublished
Dendrocorticium polygonioides (P. Karst.) M.J. Larsen & Gilb.	CBS:110.56	FR	MH857528	MH869064	[20]
	MG48	FI	HM046877	–	[46]
**ROOT:** *Punctulariopsis yunnanensis* A. Muhammad & C.L. Zhao	CLZhao 21443	CN	PP425905	PP572972	[47]
Resinicium **Parmasto phylogeny**					
Resinicium austroasianum Jia Yu, Xue W. Wang, S.L. Liu & L.W. Zhou	LWZ 20180417-5*		MW414504	MW414450	[48]
	LWZ 20191208-11		ON063691	ON063891	[49]
Resinicium bicolor (Alb.) & Schwein.) Parmasto	Miettinen 18183	FI	MF319079	MF318936	Unpublished
	AFTOL-810		DQ218310	–	[50]
Resinicium confertum Nakasone	FP-102863*	PR	DQ826538	MH877607	[51]
	He 5994	CN	MW567757	–	Unpublished
Resinicium friabile Hjortstam & Melo	LWZ 20210923-23a		MH864058	ON063692	Unpublished
	CBS:126043	NZ	MH864058	MH875513	[20]
Resinicium grandisporum G. Gruhn, Dumez & Schimann	GGMAR12-326	MQ	KY995329	–	[52]
	GGGUY13-031	GF	KY995327	–	[52]
	GGGUY13-030	GF	KY995326	–	[52]
	GGGUY13-008	GF	KY995325	–	[52]
	**ACB1491**	**CO**	**PV330475**	**PV299525**	**This study**

Resinicium lateastrocystidium Jia Yu, Xue W. Wang, S.L. Liu & L.W. Zhou (T)	LWZ 20180414-15*		MW414509	MW414455	[48]
	LWZ 20180414-13		MW414508	MW414454	[48]
Resinicium monticola Nakasone	FP-150407	JM	DQ826554	–	[51]
	FP-150355	JM	DQ826553	–	[51]
Resinicium mutabile Nakasone	GGGUY12-087	GY	KY995322	–	[52]
	GGMAR15-174	MQ	KY995330	–	[52]
Resinicium rimulosum Nakasone	BPI 878250*	JM	NR_119611	NG_064266	[51]
	KUC20131022-12	SK	KJ668464	KJ668315	Unpublished
Resinicium sacharicola (Burt.) Nakasone	GGGUY12-118	French	KY995323	–	[52]
	FP-102754	PR	DQ826547	DQ863691	[51]
Resinicium tenue Nakasone	FP-150354	JM	DQ826539	–	[51]
Schizocorticium lene (Jia Yu, Xue Wang, S.L. Liu & L.W. Zhou) L.W. Zhou	LWZ20180921-17*	–	MW414522	MW414468	[48]
	LWZ 20180921-32	–	MW414524	MW414479	[48]
Schizocorticium parvisporum Sheng H. Wu & C.L. Wei	LWZ 20210919-37a	–	ON063697	ON063761	[49]
Sidera lenis (P. Karst.) Miettinen	Miettinen 11036	FI	FN907914	FN907914	[53]
Sidera minutipora (Rodway & Cleland) Y.C. Dai, F. Wu, G.M. Gates & Rui Du	Cui 16720	AU	MN621349	MN621348	[54]
**ROOT:** *Skvortzovia qilianensis* Jia Yu, Xue W. Wang, S.L. Liu & L.W. Zhou	LWZ 20180904-20	–	ON063693	ON063893	[49]
***Trametes* phylogeny**					
Earliella scabrosa (Pers.) Gilb. & Ryvarden	PR1209	PR	JN165009	JN164793	[55]
	CIRM-BRFM 1817	US	OL685338	OL685338	[56]
	URM7788	BR	MG870412	–	[57]
	**ACB1678**	**CO**	**PV330455**	**PV299507**	**This study**
Hexagonia apiaria (Pers.) Fr.	Cui 6447	CN	KC867362	KC867481	Unpublished
H. glabra Lév.	Cui 11380	CN	KX900636	KX900685	Unpublished
	Cui 16796	AU	MK192431	MK192451	[58]
H. hirta (P. Beauv.) Fr.	Cui4051	CN	KC867359	KC867471	Unpublished

H. variegata Berk.	CBS:289.51	AR	MH856864	MH868382	[20]
	VO1754	MX	MT939262	–	Unpublished
T. betulina (L.) Pilát	HHB9942sp	US	JN164983	JN164794	[55]
T. cinnabarina (Jacq.) Fr.	AFTOL-ID 772	–	DQ411525	AY684160	Unpublished
T. conchifer (Schwein.) Pilát	FP106793sp	US	JN164988	JN164797	[55]
T. ellipsospora Ryvarden	**ACB1493**	**CO**	**PV330477**	**PV299528**	**This study**
	Cui 6259	CN	JN048767	JN048786	[59]
	Cui 8384	CN	KC848250	KC848337	Unpublished
T. hirsuta (Wulfen) Lloyd	RLG5133T	US	JN164941	JN164801	[55]
T. menziesii (Berk.) Ryvarden	**ACB1350**	**CO**	**PV330478**	**PV299529**	**This study**
	**ACB1354**	**CO**	**PV330479**	**PV299530**	**This study**
	**ACB1375**	**CO**	**PV330480**	**PV299531**	**This study**
	**ACB1378**	**CO**	**PV330481**	**PV299532**	**This study**
	**ACB1574**	**CO**	**PV330482**	**PV299533**	**This study**
	**ACB1590**	**CO**	**PV330483**	**PV299534**	**This study**
	**ACB1658**	**CO**	**PV330484**	**PV299535**	**This study**
	**ACB1661**	**CO**	**PV330485**	**PV299536**	**This study**
	**ACB1662**	**CO**	**PV330486**	**PV299537**	**This study**
	Cui 16808	AU	OK642207	OK642262	Unpublished
	TJV 93-213sp	US	OQ539599	–	[60]
	Dai 6782	–	KC848289	KC848374	Unpublished
	Zhou 72	–	KC848328	KC848412	Unpublished
T. polyzona (Pers.) Justo	**ACB1475**	**CO**	**PV330487**	**PV299538**	**This study**
	**ACB1492**	**CO**	**PV330488**	**PV299539**	**This study**
	**ACB1585**	**CO**	**PV330489**	**PV299540**	**This study**
	BKW004	GA	JN164978	JN164790	[55]
T. sanguinea (Klotzsch) Pat.	**ACB1476**	**CO**	**PV330474**	**PV299524**	**This study**
	PRSC95	PR	JN164982	JN164795	[55]

T. versicolor (L.) Lloyd	FP-135156-Sp	US	KJ995937	–	Unpublished
T. villosa (Sw.) Kreisel	FP71974R	US	JN164969	JN164810	[55]
**ROOT:** *H. cucullata* (Mont.) Murrill	CulTENN11221	AR	AF516600	AJ488124	[11]

References

1. Zhou, M.; Wang, C.G.; Wu, Y. Da; Liu, S.; Yuan, Y. Two New Brown Rot Polypores from Tropical China. *MycoKeys* 2021, *82*, 173–197, doi:10.3897/MYCOKEYS.82.68299.

2. Li, H.J.; Han, M.L.; Cui, B.K. Two New Fomitopsis Species from Southern China Based on Morphological and Molecular Characters. *Mycol Prog* 2013, *12*, 709–718, doi:10.1007/s11557-012-0882-2.

3. Ortiz-Santana, B.; Lindner, D.L.; Miettinen, O.; Justo, A.; Hibbett, D.S. A Phylogenetic Overview of the Antrodia Clade (Basidiomycota, Polyporales). *Mycologia* 2013, *105*, 1391–1411, doi:10.3852/13-051.

4. Liu, S.; Song, C.G.; Cui, B.K. Morphological Characters and Molecular Data Reveal Three New Species of Fomitopsis (Basidiomycota). *Mycol Prog* 2019, *18*, 1317–1327, doi:10.1007/s11557-019-01527-w.

5. Spirin, V.; Runnel, K.; Vlasák, J.; Viner, I.; Barrett, M.D.; Ryvarden, L.; Bernicchia, A.; Rivoire, B.; Ainsworth, A.M.; Grebenc, T.; et al. The Genus Fomitopsis (Polyporales, Basidiomycota) Reconsidered. *Stud Mycol* 2024, *107*, 149–249, doi:10.3114/sim.2024.107.03.

6. Han, M.L.; Chen, Y.Y.; Shen, L.L.; Song, J.; Vlasák, J.; Dai, Y.C.; Cui, B.K. Taxonomy and Phylogeny of the Brown-Rot Fungi: Fomitopsis and Its Related Genera. *Fungal Divers* 2016, *80*, 343–373, doi:10.1007/s13225-016-0364-y.

7. Zhou, J.L.; Zhu, L.; Chen, H.; Cui, B.K. Taxonomy and Phylogeny of Polyporus Group Melanopus (Polyporales, Basidiomycota) from China. *PLoS One* 2016, *11*, doi:10.1371/journal.pone.0159495.

8. Sathiya Seelan, J.S.; Justo, A.; Nagy, L.G.; Grand, E.A.; Redhead, S.A.; Hibbett, D. Phylogenetic Relationships and Morphological Evolution in Lentinus, Polyporellus and Neofavolus, Emphasizing Southeastern Asian Taxa. *Mycologia* 2015, *107*, 460–474, doi:10.3852/14-084.

9. Grand, E.A.; Hughes, K.W.; Petersen, R.H. Relationships within Lentinus Subg. Lentinus (Polyporales, Agaricomycetes), with Emphasis on Sects. Lentinus and Tigrini. *Mycol Prog* 2011, *10*, 399–413, doi:10.1007/s11557-010-0711-4.

10. Sotome, K.; Hattori, T.; Ota, Y.; Kakishima, M. Second Report of Polyporus Longiporus and Its Phylogenetic Position. *Mycoscience* 2009, *50*, 415–420, doi:10.1007/s10267-009-0506-0.

11. Krueger, D. Trace: Tennessee Research and Creative Exchange Monographic Studies in the Genus Polyporus (Basidiomycotina) Recommended Citation, University of Tennessee, Knoxville: Tennessee, 2002.

12. Matozaki, T.; Hattori, T.; Kuwahara, T.; Boonlue, S.; Maekawa, N.; Nakagiri, A.; Endo, N.; Sotome, K. *First Report of Polyporus Ciliatus in Japan, and Taxonomic Re-Evaluation of Synonymous Species Described from Japan*; 2019; Vol. 27;.

13. Li, H.J.; Cui, B.K.; Dai, Y.C. Taxonomy and Multi-Gene Phylogeny of Datronia (Polyporales, Basidiomycota). *Persoonia: Molecular Phylogeny and Evolution of Fungi* 2014, *32*, 170–182, doi:10.3767/003158514X681828.

14. Jang, Y.; Jang, S.; Min, M.; Hong, J.H.; Lee, H.; Lee, H.; Lim, Y.W.; Kim, J.J. Comparison of the Diversity of Basidiomycetes from Dead Wood of the Manchurian Fir (Abies Holophylla) as Evaluated by Fruiting Body Collection, Mycelial Isolation, and 454 Sequencing. *Microb Ecol* 2015, *70*, 634–645, doi:10.1007/s00248-015-0616-5.

15. Matozaki, T.; Hattori, T.; Maekawa, N.; Nakagiri, A.; Ishikawa, N.K.; Sotome, K. Hirticrusta Gen. Nov. Segregated from Neofomitella in Polyporaceae (Polyporales). *Mycoscience* 2020, *61*, 240–248, doi:10.1016/j.myc.2020.03.007.

16. Henrik Nilsson, R.; Hallenberg, N. Phylogeny of the Hypochnicium Punctulatum Complex as Inferred from ITS Sequence Data. *Mycologia* 2003, *95*, 54–60, doi:10.1080/15572536.2004.11833131.

17. Paulus, B.; Nilsson, H.; Hallenberg, N. Phylogenetic Studies in Hypochnicium (Basidiomycota), with Special Emphasis on Species from New Zealand. *N Z J Bot* 2007, *45*, 139–150, doi:10.1080/00288250709509709.

18. Telleria, M.T.; Dueñas, M.; Melo, I.; Hallenberg, N.; Martín, M.P. A Re-Evaluation of Hypochnicium (Polyporales) Based on Morphological and Molecular Characters. *Mycologia* 2010, *102*, 1426–1436, doi:10.3852/09-242.

19. Maekawa, N.; Sugawara, R.; Kogi, H.; Norikura, S.; Sotome, K.; Endo, N.; Nakagiri, A.; Ushijima, S. Hypochnicium Sensu Lato (Polyporales, Basidiomycota) from Japan, with Descriptions of a New Genus and Three New Species. *Mycoscience* 2023, *64*, 19–34, doi:10.47371/mycosci.2022.10.001.

20. Vu, D.; Groenewald, M.; de Vries, M.; Gehrmann, T.; Stielow, B.; Eberhardt, U.; Al-Hatmi, A.; Groenewald, J.Z.; Cardinali, G.; Houbraken, J.; et al. Large-Scale Generation and Analysis of Filamentous Fungal DNA Barcodes Boosts Coverage for Kingdom Fungi and Reveals Thresholds for Fungal Species and Higher Taxon Delimitation. *Stud Mycol* 2019, *92*, 135–154, doi:10.1016/j.simyco.2018.05.001.

21. Justo, A.; Miettinen, O.; Floudas, D.; Ortiz-Santana, B.; Sjökvist, E.; Lindner, D.; Nakasone, K.; Niemelä, T.; Larsson, K.H.; Ryvarden, L.; et al. A Revised Family-Level Classification of the Polyporales (Basidiomycota). *Fungal Biol* 2017, *121*, 798–824, doi:10.1016/j.funbio.2017.05.010.

22. Larsson, K.H. Molecular Phylogeny of Hyphoderma and the Reinstatement of Peniophorella. *Mycol Res* 2007, *111*, 186–195, doi:10.1016/j.mycres.2006.10.002.

23. Menkis, A.; Allmer, J.; Vasiliauskas, R.; Lygis, V.; Stenlid, J.; Finlay, R. Ecology and Molecular Characterization of Dark Septate Fungi from Roots, Living Stems, Coarse and Fine Woody Debris. *Mycol Res* 2004, *108*, 965–973, doi:10.1017/S0953756204000668.

24. Jang, Y.; Lee, S.W.; Lim, Y.W.; Lee, J.S.; Hallenberg, N.; Kim, J.J. Hypochnicium Pini, a New Corticioid Basidiomycete in East Asia. *Mycotaxon* 2013, *124*, 209–217, doi:10.5248/124.209.

25. Elisabet Sjökvist 1, E.L.U.E.L.R.K.-H.L. Stipitate Stereoid Basidiocarps Have Evolved Multiple Times. *Mycologia* 2012, *104*, 1046–1055.

26. Mueller, G.M.; Wu, Q.X.; Huang, Y.Q.; Guo, S.Y.; Aldana-Gomez, R.; Vilgalys, R. Assessing Biogeographic Relationships between North American and Chinese Macrofungi. In Proceedings of the Journal of Biogeography; 2001; Vol. 28, pp. 271–281.

27. Qin, J.; Horak, E.; Popa, F.; Rexer, K.H.; Kost, G.; Li, F.; Yang, Z.L. Species Diversity, Distribution Patterns, and Substrate Specificity of Strobilurus. *Mycologia* 2018, *110*, 584–604, doi:10.1080/00275514.2018.1463064.

28. Antonín, V.; Ryoo, R.; Ka, K.H.; Sou, H.D. Three New Species of Crinipellis and One New Variety of Moniliophthora (Basidiomycota, Marasmiaceae) Described from the Republic of Korea. *Phytotaxa* 2014, *170*, 86–102, doi:10.11646/phytotaxa.170.2.2.

29. Bodensteiner, P.; Binder, M.; Moncalvo, J.M.; Agerer, R.; S. Hibbett, D. Phylogenetic Relationships of Cyphelloid Homobasidiomycetes. *Mol Phylogenet Evol* 2004, *33*, 501–515, doi:10.1016/j.ympev.2004.06.007.

30. Antonín, V.; Hosaka, K.; Kolařík, M. Taxonomy and Phylogeny of Paramarasmius Gen. Nov. and Paramarasmius Mesosporus, a Worldwide Distributed Fungus with a Strict Ecological Niche. *Plant Biosyst* 2023, *157*, 286–293, doi:10.1080/11263504.2022.2100503.

31. Zhao, D.L.; Wang, D.; Tian, X.Y.; Cao, F.; Li, Y.Q.; Zhang, C.S. Anti-Phytopathogenic and Cytotoxic Activities of Crude Extracts and Secondary Metabolites of Marine-Derived Fungi. *Mar Drugs* 2018, *16*, doi:10.3390/md16010036.

32. de Oliveira, J.J.S.; Moncalvo, J.M.; Margaritescu, S.; Capelari, M. Phylogenetic and Morphological Analyses of Species of Marasmius Sect. Marasmius from the Atlantic Rainforest, Brazil. *Plant Systematics and Evolution* 2020, *306*, doi:10.1007/s00606-020-01659-7.

33. Chen, C.C.; Chen, C.Y.; Wu, S.H. Species Diversity, Taxonomy and Multi-Gene Phylogeny of Phlebioid Clade (Phanerochaetaceae, Irpicaceae, Meruliaceae) of Polyporales. *Fungal Divers* 2021, *111*, 337–442, doi:10.1007/s13225-021-00490-w.

34. Zhao, Y.N.; He, S.H.; Nakasone, K.K.; Wasantha Kumara, K.L.; Chen, C.C.; Liu, S.L.; Ma, H.X.; Huang, M.R. Global Phylogeny and Taxonomy of the Wood-Decaying Fungal Genus Phlebiopsis (Polyporales, Basidiomycota). *Front Microbiol* 2021, *12*, 1–20, doi:10.3389/fmicb.2021.622460.

35. Xavier de Lima, V.; Lira, C. de S.; Chikowski, R. dos S.; Santos, C.; Lima, N.; Gibertoni, T.B. Additions to Neotropical Stereoid Fungi (Polyporales, Basidiomycota): One New Species of Lopharia and One New Combination in Phlebiopsis. *Mycol Prog* 2020, *19*, 31–40, doi:10.1007/s11557-019-01538-7.

36. Miettinen, O.; Spirin, V.; Vlasák, J.; Rivoire, B.; Stenroos, S.; Hibbett, D.S. Polypores and Genus Concepts in Phanerochaetaceae (Polyporales, Basidiomycota). *MycoKeys* 2016, *17*, 1–46, doi:10.3897/mycokeys.17.10153.

37. Floudas, D.; Hibbett, D.S. Revisiting the Taxonomy of Phanerochaete (Polyporales, Basidiomycota) Using a Four Gene Dataset and Extensive ITS Sampling. *Fungal Biol* 2015, *119*, 679–719, doi:10.1016/j.funbio.2015.04.003.

38. Dong, J.H.; Li, Q.; Yuan, Q.; Luo, Y.X.; Zhang, X.C.; Dai, Y.F.; Zhou, Q.; Liu, X.F.; Deng, Y.L.; Zhou, H.M.; et al. Species Diversity, Taxonomy, Molecular Systematics and Divergence Time of Wood-Inhabiting Fungi in Yunnan-Guizhou Plateau, Asia. *Mycosphere* 2024, *15*, 1110–1293, doi:10.5943/MYCOSPHERE/15/1/10.

39. Wu, S.H.; Nilsson, H.R.; Chen, C.T.; Yu, S.Y.; Hallenberg, N. The White-Rotting Genus Phanerochaete Is Polyphyletic and Distributed throughout the Phleboid Clade of the Polyporales (Basidiomycota). *Fungal Divers* 2010, *42*, 107–118, doi:10.1007/s13225-010-0031-7.

40. Chen, C.C.; Chen, C.Y.; Wu, S.H. Species Diversity, Taxonomy and Multi-Gene Phylogeny of Phlebioid Clade (Phanerochaetaceae, Irpicaceae, Meruliaceae) of Polyporales. *Fungal Div.* 2021, *111*, 337–442, doi:doi: 10.1007/s13225-021-00490-w.

41. Mao, W.L.; Wu, Y. Da; Liu, H.G.; Yuan, Y.; Dai, Y.C. A Contribution to Porogramme (Polyporaceae, Agaricomycetes) and Related Genera. *IMA Fungus* 2023, *14*, doi:10.1186/s43008-023-00110-z.

42. Yuan, H.S.; Wan, X.Z. Morphological and ITS RDNA-Based Phylogenetic Identification of Two New Species in Tinctoporellus. *Mycol Prog* 2012, *11*, 947–952, doi:10.1007/s11557-012-0810-5.

43. Wei, C.L.; Chen, C.C.; He, S.H.; Wu, S.H. Dendrocorticiopsis Orientalis Gen. et Sp. Nov. of the Punctulariaceae (Corticiales, Basidiomycota) Revealed by Molecular Data. *MycoKeys* 2022, *90*, 19–30, doi:10.3897/mycokeys.90.84562.

44. Acero, D.; Khan, F.S.T.; Medina-Ortiz, A.J.; Rivero-Cruz, I.; Raja, H.A.; Flores-Bocanegra, L.; Fajardo-Hernández, C.A.; Wan, B.; Franzblau, S.G.; Hematian, S.; et al. New Terpenoids from the Corticioid Fungus Punctularia Atropurpurascens and Their Antimycobacterial Evaluation. *Planta Med* 2022, *88*, 729–734, doi:10.1055/a-1786-8072.

45. Guan, Q.-X.W.Z. and C.-L.Z. A New Species of Punctularia (Punctulariaceae, Basidiomycota) from Southwest China. *Phytotaxa* 2021, *489*, 285–292, doi:https://doi.org/10.11646/phytotaxa.489.3.5.

46. Ghobad-Nejhad, M.; Nilsson, R.H.; Hallenberg, N. Phylogeny and Taxonomy of the Genus Vuilleminia (Basidiomycota) Based on Molecular and Morphological Evidence, with New Insights into Corticiales. *Taxon* 2010, *59*, 1519–1534, doi:10.1002/tax.595016.

47. Muhammad, A.; Deng, Y.; Dai, Y.; Su, J.; Zhao, C. Phylogenetic and Taxonomic Evidence Reveal Punctulariopsis Yunnanensis Sp. Nov. (Punctulariaceae, Basidiomycota) from Southwest China. *Phytotaxa* 2024, *663*, 59–68, doi:10.11646/phytotaxa.663.2.1.

48. Yu, J.; Wang, X.W.; Liu, S.L.; Shen, S.; Zhou, L.W. Taxonomy and Phylogeny of Resinicium Sensu Lato from Asia-Pacific Revealing a New Genus and Five New Species (Hymenochaetales, Basidiomycota). *IMA Fungus* 2021, *12*, doi:10.1186/s43008-021-00071-1.

49. Wang, X.W.; Liu, S.L.; Zhou, L.W. An Updated Taxonomic Framework of Hymenochaetales (Agaricomycetes, Basidiomycota). *Mycosphere* 2023, *14*, 452–496, doi:10.5943/mycosphere/14/1/6.

50. Brandon Matheny, P.; Wang, Z.; Binder, M.; Curtis, J.M.; Lim, Y.W.; Henrik Nilsson, R.; Hughes, K.W.; Hofstetter, V.; Ammirati, J.F.; Schoch, C.L.; et al. Contributions of Rpb2 and Tef1 to the Phylogeny of Mushrooms and Allies (Basidiomycota, Fungi). *Mol Phylogenet Evol* 2007, *43*, 430–451, doi:10.1016/j.ympev.2006.08.024.

51. Nakasone, K.K. Morphological and Molecular Studies on Resinicium s. Str. *Canadian Journal of Botany* 2007, *85*, 420–436, doi:10.1139/B07-035.

52. Gruhn, G.; Dumez, S.; Moreau, P.A.; Roy, M.; Morreale, O.; Schimann, H.; Courtecuisse, R. The Genus Resinicium in French Guiana and the West Indies: A Morphological and Molecular Survey, Revealing Resinicium Grandisporum Sp. Nov. *Cryptogam Mycol* 2017, *38*, 469–483, doi:10.7872/crym/v38.iss4.2017.469.

53. Miettinen, O.; Larsson, K.H. Sidera, a New Genus in Hymenochaetales with Poroid and Hydnoid Species. *Mycol Prog* 2011, *10*, 131–141, doi:10.1007/s11557-010-0682-5.

54. Du, R.; Wu, F.; Gate, G.M.; Dai, Y.C.; Tian, X.M. Taxonomy and Phylogeny of Sidera (Hymenochaetales, Basidiomycota): Four New Species and Keys to Species of the Genus. *MycoKeys* 2020, *68*, 115–135, doi:10.3897/MYCOKEYS.68.53561.

55. Justo, A.; Hibbett, D.S. Phylogenetic Classification of Trametes (Basidiomycota, Polyporales) Based on a Five-Marker Dataset. *Taxon* 2011, *60*, 1567–1583, doi:10.1002/tax.606003.

56. Hage, H.; Miyauchi, S.; Virágh, M.; Drula, E.; Min, B.; Chaduli, D.; Navarro, D.; Favel, A.; Norest, M.; Lesage-Meessen, L.; et al. Gene Family Expansions and Transcriptome Signatures Uncover Fungal Adaptations to Wood Decay. *Environ Microbiol* 2021, *23*, 5716–5732, doi:10.1111/1462-2920.15423.

57. de Pádua, A.P.S.L.; Freire, K.T.L. de S.; de Oliveira, T.G.L.; Silva, L.F. da; Araújo-Magalhães, G.R.; Agamez-Montalvo, G.S.; da Silva, I.R.; Bezerra, J.D.P.; de Souza-Motta, C.M. Fungal Endophyte Diversity in the Leaves of the Medicinal Plant Myracrodruon Urundeuva in a Brazilian Dry Tropical Forest and Their Capacity to Produce L-Asparaginase. *Acta Bot Brasilica* 2019, *33*, 39–49, doi:10.1590/0102-33062018abb0108.

58. Ji, X.; Wu, D.M.; Song, C.G.; Liu, S.; Si, J.; Cui, B.K. Two New Neofomitella Species (Polyporaceae, Basidiomycota) Based on Morphological and Molecular Evidence. *Mycol Prog* 2019, *18*, 593–602, doi:10.1007/s11557-019-01472-8.

59. Zhao, C.L.; Cui, B.K.; Steffen, K.T. Yuchengia, a New Polypore Genus Segregated from Perenniporia (Polyporales) Based on Morphological and Molecular Evidence. *Nord J Bot* 2013, *31*, 331–338, doi:10.1111/j.1756-1051.2012.00003.x.

60. Yu, J.; Lai, J.; Neal, B.M.; White, B.J.; Banik, M.T.; Dai, S.Y. Genomic Diversity and Phenotypic Variation in Fungal Decomposers Involved in Bioremediation of Persistent Organic Pollutants. *Journal of Fungi* 2023, *9*, doi:10.3390/jof9040418.
